# Efecto de la contaminación por lípidos exógenos en la gasometría

**DOI:** 10.1515/almed-2024-0061

**Published:** 2024-05-08

**Authors:** Giuseppe Lippi, Laura Pighi, Gian Luca Salvagno, Elena Tiziani, Maria Elena Castellini, Roberta Ferraro, Brandon M. Henry

**Affiliations:** Section Departamento de Bioquímica Clínica y FAcultad de Medicina, 19051 Universidad de Verona, Verona, Italia; Laboratorio Clínico, División de Nefrología e Hipertensión, 2518 Cincinnati Children’s Hospital Medical Center, Cincinnati, OH, Estados Unidos

**Keywords:** análisis de gases en sangre, errores, jeringa, lipidos, interferencia

## Abstract

**Objetivos:**

El objetivo del presente estudio es investigar los efectos de la contaminación de sangre venosa por una solución de lípidos sobre diferentes parámetros, determinados en un moderno analizador de gases en sangre.

**Métodos:**

Se extrajo sangre venosa de 17 profesionales sanitarios (46±11 años; 53 % mujeres) y se introdujo en tres jeringas de gasometría, que contenían una solución de lípidos al 0 %, 5 % y 10 %. En los 15 minutos siguientes a la extracción de la muestra, se realizó la gasometría con un analizador GEM Premier 5000. Los triglicéridos e índices séricos se analizaron en el dispositivo COBAS C702 de Roche.

**Resultados:**

La concentración de triglicéridos aumentó de 1.0±0.3 mmol/L en la jeringa de gasometría no contaminada a 39,4±7,8 y 65,3±14,4 mmol/L (ambas p<0.001) en las jeringas con contaminación por lípidos al 5 % y al 10 %. Como consecuencia, los valores de los índices lipémico y hemolítico aumentaron. Observamos una variación estadísticamente significativa en todos los analitos, excepto en el hematocrito y la COHb en la jeringa con lípidos al 5 %, siendo COHb el único analito que no varió en la jeringa con lípidos al 10 %. Los valores de pO_2_, SO_2_ y lactato aumentaron significativamente a partir del 5 % de contaminación por lípidos, mientras que se produjo un descenso de los valores de pH, pCO_2_, sodio, potasio, cloruro, calcio ionizado, glucosa, hematocrito (contaminación al 10 %), hemoglobina y MetHB. Todas estas variaciones, excepto en el caso del lactato y la CoHb, superaron sus especificaciones de calidad relativa.

**Conclusiones:**

La hiperlipidemia artefactual causada por la contaminación por lípidos exógenos podría tener un impacto clínicamente significativo en los resultados de la gasometría. Se debe instar a los fabricantes de analizadores de gases en sangre a que desarrollen nuevos instrumentos que incluyan la determinación de índices séricos.

## Introducción

El análisis de gases en sangre es una prueba indispensable en el diagnóstico de enfermedades metabólicas graves, desequilibrio ácido–base, estado de oxigenación anormal y deterioro general de la función respiratoria [1]. El uso clínico de esta prueba aumentó notablemente durante la pandemia de enfermedad por coronavirus 2019 (COVID-19), debido a las frecuentes alteraciones ácido-base que presentaban los pacientes con infección por SARS-CoV-2 grave, así como debido a su estrecha relación con el pronóstico a corto y medio plazo [2]. Los analizadores de gases en sangre que se emplean actualmente abarcan una amplia gama de analitos mensurables, además de parámetros tradicionales como el pH, la presión parcial de oxígeno (pO_2_), la saturación de oxígeno (SO_2_) y la presión parcial de dióxido de carbono (pCO_2_), incluyendo los electrolitos (potasio, sodio y cloruro), la hemoglobina total (tHb) y el hematocrito (Hct), el calcio ionizado (iCa^2+^), la glucosa, el lactato, la carboxihemoglobina (COHb) y la metahemoglobina (MetHb) [3]. El uso de analizadores de gases en sangre es especialmente adecuado para áreas médicas clave como las salas de urgencias, las unidades de cuidados intensivos (tanto de adultos como pediátricas), las unidades de diálisis y los quirófanos, donde es esencial contar con una evaluación rápida y exacta de parámetros metabólicos y respiratorios básicos, con el fin de garantizar un tratamiento y una asistencia adecuada [4].

Para que sean de utilidad en la toma de decisiones clínicas, los resultados de las gasometrías deben ser exactos y fiables, con el objeto de evitar o minimizar la posible influencia de algunas variables preanalíticas en las determinaciones [5, 6]. Al igual que en las pruebas de laboratorio convencionales, en la gasometría, la calidad de todo el proceso analítico se puede ver afectada por algunos problemas pre analíticos. La lipemia es una de las principales causas de preocupación, ya que tiene un impacto significativo, causando desviaciones en algunos parámetros de química clínica, hematología y hemostasia, tal como se ha descrito profusamente en la literatura [7].

Aunque la guía clínica C46A2 aprobada recientemente por el Clinical and Laboratory Standards Institute (CLSI) para el análisis de gases en sangre establece que únicamente la turbidez de la muestra, como la causada por la hiperlipemia o la administración de emulsiones lipídicas, puede afectar a la evaluación de las fracciones de tHb y hemoglobina [8], estudios recientes sugieren que la lipemia podría comprometer la calidad de la gasometría, al alterar los valores de otros analitos que pueden medirse con los analizadores de gases en sangre actuales [9]. Por todo ello, se llevó a cabo el presente estudio para analizar los efectos de la contaminación de sangre venosa con una solución lipídica en una amplia muestra de parámetros determinados en un analizador de gases en sangre moderno.

## Materiales y métodos

Se extrajo sangre venosa a 17 miembros del personal (media de edad; 46±11 años; 53 % mujeres) del Servicio de Medicina de Laboratorio del Hospital Universitario de Verona (Italia). Se localizó una vena accesible en la parte superior de los antebrazos y se realizó una punción con una aguja mariposa 21G×3/4” (0,8×20 mm) (Safety Blood Collection Set, Gemtier Medical, Shangai, China), conectada inicialmente a un tubo de heparina de litio de 3,5 mL al vacío (Vacutest Kima, Padua, Italia) para descartar la presencia de aire residual en el tubo. A continuación, se aspiró sangre venosa consecutivamente en tres jeringas de gasometría heparinizadas de 1,0 mL, 0,5×16 mm, hasta alcanzar su volumen nominal (Arterial Blood Sampling Kit, Smiths Medical ASD IN, Minneapolis, MN, EE.UU). La primera jeringa se dejó vacía previamente a la extracción de sangre, mientras que la segunda y la tercera fuero precargadas con 0,05 y 0,10 mL de una solución de lípidos (Lipidem 200 mg/mL; B. Braun Medical, Sheffield, Reino Unido) (Tabla 1), resultando en una contaminación del 5 % y 10 % de la muestra final. La composición concreta de dicha emulsión lipídica es la siguiente: 10 % de triglicéridos de cadena media, 8 % de aceite de soja (triglicéridos de cadena larga, principalmente omega-6) y 2 % de ácidos grasos omega-3 (triglicéridos de cadena larga), con lo que el contenido medio de triglicéridos es del 20 %. El intervalo suele estar comprendido entre 0,7 y 1,5 lípidos por kg de peso corporal y día. Inmediatamente después de la extracción, se taparon las tres jeringas y se mezclaron girándolas suavemente entre las palmas de las manos durante aproximadamente 20 segundos, para que se mezclaran correctamente la sangre venosa, la solución de litio-heparina y la solución lipídica presentes en la segunda y tercera jeringa. No había espacio muerto en ninguna de las jeringas, lo que excluye la posible presencia de aire ambiente.

**Figura 1:**
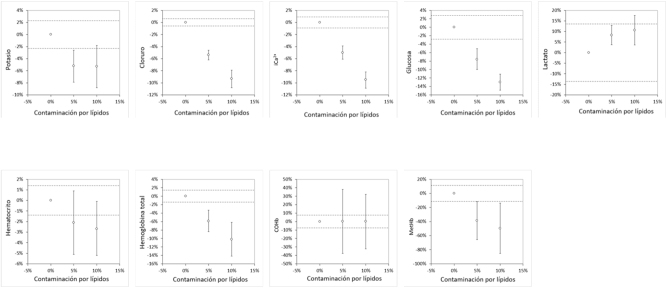
Impacto de la contaminación de la sangre venosa con una solución de lípidos (5 % y 10 % de contaminación) en la gasometría. Los resultados se presentan como desviación media (e intervalo de confianza del 95 %; IC 95 %) frente a la jeringa de gasometría no contaminada de referencia. Las líneas discontinuas representan las especificaciones de calidad (±) para cada analito analizado. ppCO_2_, presión parcial de dióxido de carbono; pO_2_, presión parcial de oxígeno; sO_2_, saturación de oxígeno; iCa^2+^, calcio ionizado; COHb, carboxihemoglobina; MetHb, metahemoglobina.

Las gasometrías se realizaron transcurridos entre 12 y 15 minutos de la extracción y empleando el mismo analizador de gases en sangre, así como el mismo lote de cassetes de prueba (GEM Premier 5000, Instrumentation Laboratory, Monza, Italia). La concentración de triglicéridos y los índices séricos se midieron en un analizador COBAS C702 de Roche (Roche Diagnostics, Basel, Suiza) tras separar el plasma mediante centrifugación. Los resultados de las determinaciones se presentan como medias y desviaciones estándar (DE). La desviación entre la jeringa de referencia (esto es, la jeringa sin contaminación por lípidos) y las que contenían las soluciones con 0,05 y 0,10 mL de lípidos, se expresa como la variación media de los resultados de las pruebas que superaban las especificaciones de calidad sugeridas por Kuster et al. [10] (Tabla 2). La significación de las variaciones de concentración de los analitos debidas a la presencia de la solución de lípidos se evaluó con la prueba *t* de Student para muestras relacionadas, mientras que la desviación porcentual con respecto a la jeringa de gasometría no contaminada de referencia se calculó con el método de Bland y Altman. La significación estadística se estableció en p<0,05. Todos los análisis estadísticos se realizaron con el programa Analyse-it (Analyse-it Software Ltd, Leeds, Reino Unido).

Todos los sujetos reclutados para el presente estudio firmaron un consentimiento informado previamente a su inclusión. El estudio se realizó con ajuste a los principios de la Declaración de Helsinki y la legislación aplicable, habiendo sido aprobado por el Comité de Ética del Hospital Universitario de Verona (código de aprobación: 970CESC; 20 de julio de 2016).

## Resultados

En la Tabla 2 se muestra un resumen de los resultados de nuestro estudio. La concentración media de triglicéridos aumentó de 1,0±0,3 mmol/L en el plasma de la jeringa de gasometría no contaminada a 39,4±7,8 mmol/L (p<0,001) en la jeringa con contaminación por una solución de lípidos al 5 %, y hasta 65,3±14,4 mmol/L (p<0,001) en la jeringa con una solución de lípidos al 10 %. Como consecuencia, el índice lipémico (Índice L) también aumentó de 10,6±3,9 en la jeringa de gasometría no contaminada, a 679,2±164,0 (p<0,001) y 1209,8±346,1 (p<0,001) en aquellas con un 5 % y 10 % de lípidos. Curiosamente, el índice ictérico (índice I) no varió significativamente en las tres jeringas, mientras que el índice de hemólisis (índice H) aumentó significativamente de 6,1±3,5 en la jeringa no contaminada, a 23,2±6,0 (p<0,001) y 43,9±20,7 (p<0,001) en las jeringas con contaminación lipídica al 5 % y 10 %.

**Table 1:** Protocolo del estudio para evaluar el impacto de la contaminación de sangre venosa con una solución lipídica en la gasometría.

Jeringa	Volume de llenado de sangre	Solución lipídica	Fracciones de sangre y lípidos
1^a^ jeringa	1,0 mL	0 mL	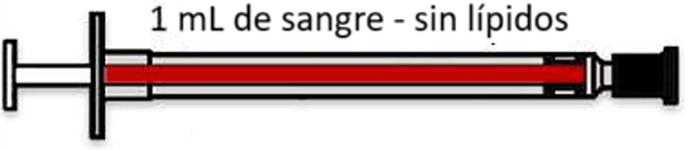
2^a^ jeringa	0,95 mL	0,05 mL	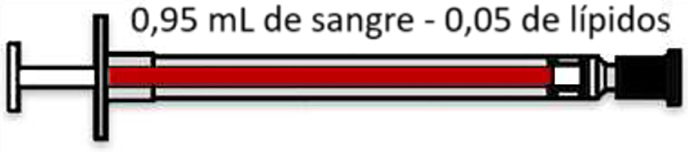
3^a^ jeringa	0,90 mL	0,10 mL	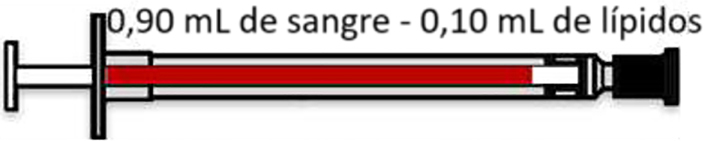

**Tabla 2:** Impacto de la contaminación de sangre venosa con una solución lipídica (5 % y 10 %) en la gasometría. Los resultados de las pruebas se presentan como medias y desviaciones estándar (DE).

Analito	Especifica ciones de calidad	Imprecisión intraensayo^a^	Sin contaminación	Contaminación con lípidos al 5 %		Contaminación con lípidos al 10 %	
			Valor	Valor	Valor p^b^	Valor	Valor p^b^
pH	±1,5 %	±0,1 %	7,35±0,02	7,34±0,02	0,003	7,33±0,02	0,001
pCO_2_, mmHg	±2,4 %	±1,4 %	49,0±4,9	46,5±4,4	<0,001	44,9±3,4	<0,001
pO_2_, mmHg	±1,5 %	±1,7 %	31,1±10,3	34,4±11,7	0,029	36,6±12,2	0,020
SO_2_, %	±1,5 %	±1,2 %	51,7±17,7	57,8±16,6	0,018	61,3±16,9	0,019
Sodio, mmol/L	±0,3 %	±0,3 %	136,9±0,9	130,2±2,5	<0,001	124,9±3,9	<0,001
Potasio, mmol/L	±2,3 %	±1,0 %	4,33±0,32	4,11±0,27	0,001	4,10±0,22	0,005
Cloruro, mmol/L	±0,6 %	±0,4 %	103,2±1,0	97,8±1,7	<0,001	94,0±2,9	<0,001
iCa^2+^, mmol/L	±0,9 %	±1,4 %	1,26±0,06	1,20±0,05	<0,001	1,15±0,06	<0,001
Glucosa, mmol/L	±2,8 %	±2,5 %	5,5±0,6	5,1±0,6	<0,001	4,8±0,5	<0,001
Lactato, mmol/L	±13,6 %	±7,5 %	1,11±0,25	1,20±0,27	0,001	1,22±0,24	0,005
Hct	±1,4 %	±3,6 %	45,1±4,0	44,2±4,5	0,162	43,9±4,1	0,041
tHb, g/L	±1,4 %	±2,0 %	148±15	140±14	<0,001	134±15	<0,001
COHb, %	±7,5 %	±43,7 %	1,57±0,96	1,61±0,99	0,937	1,60±1,16	0,996
MetHb, %	±11,3 %	±9,3 %	0,89±0,22	0,65±0,29	0,008	0,61±0,37	0,001
Triglicéridos, mmol/L	–	–	1,0±0,3	39,4±7,8	<0,001	65,3±14,4	<0,001
Índice H	–	–	6,1±3,5	23,2±6,0	<0,001	43,9±20,7	<0,001
Índice L	–	–	10,6±3,9	679,2±164,0	<0,001	1209,8±346,1	<0,001
Índice I	–	–	19,8±7,7	20,4±8,2	0,491	20,7±8,4	0,364

^a^Imprecisión intraensayo del analizador calculada sobre muestras venosas ordinarias; ^b^en comparación con la jeringa de gasometría de referencia sin contaminación lipídica. IC 95 %, intervalo de confianza del 95 %; pCO_2_, presión parcial de dióxido de carbono; pO_2_, presión parcial de oxígeno; sO_2_, saturación de oxígeno; iCa^2+^, calcio ionizado; Hct, hematocrito; tHb, hemoglobina total; COHb, carboxihemoglobina; MetHb, metahemoglobina.

En cuanto a los parámetros de la gasometría, se observó una variación estadísticamente significativa en todos los analitos, excepto en el Hct y la COHb, en la jeringa que contenía una solución de lípidos al 5 %, mientras que la COHb fue la única que no varió significativamente en la jeringa con una solución de lípidos al 10 %. Tal como se muestra en la Tabla 2 y en la Figura 1, se observaron aumentos significativos a partir de la contaminación con lípidos al 5 %, en las concentraciones de pO_2_, SO_2_ y lactato, mientras que se produjo una disminución de los valores de pH, pCO_2_, sodio, potasio, cloruro, iCa^2+^, glucosa, Hct (sólo en la jeringa que contenía lípidos al 10 %), tHb y MetHB. Cuando comparamos dichas variaciones con las especificaciones de calidad [10], la totalidad de las variaciones, excepto el lactato y la COHb, superaban sus límites relativos, (Figura 1).

## Discusión

El análisis de gases en sangre permite obtener información clínica importante sobre el metabolismo, el equilibrio ácido–base y el estado de oxigenación, que son cruciales para el diagnóstico y el tratamiento de diversas patologías, especialmente las relacionadas con trastornos respiratorios y metabólicos [11]. De este modo, es esencial garantizar un alto nivel de calidad a lo largo de todo el proceso de análisis de gases en sangre, desde la extracción de la muestra hasta la comunicación de los resultados, ya que unos resultados poco fiables pueden perjudicar al cuidado del paciente [12]. En consecuencia, los profesionales sanitarios deberían velar por garantizar la calidad del proceso, asegurándose en primer lugar de que la extracción de sangre se realiza correctamente y de que su contenido es representativo de las condiciones *in vivo* del paciente.

Aunque en multitud de pruebas analíticas se suelen adoptar estas precauciones (p.ej. química clínica, inmunoquímica, hemostasia, hematología), existen escasos estudios sobre la posible interferencia por contaminación de sangre venosa por la presencia de lípidos en las jeringas de gasometría, a pesar de que esta se podría estar produciendo con relativa frecuencia. Un estudio reciente realizado por Liu et al. reveló que casi el 6 % de todas las muestras para gasometría obtenidas en su centro presentaban niveles clínicamente significativos de índice L [13]. En un estudio previo en el que se analizó la presencia de hemólisis, lipemia e ictericia en todas las muestras de gasometría, Salvagno y col observaron una presencia mucho más frecuente de muestras lipémicas, siendo esta de casi el doble (p.ej. 11 %) [14]. Por lo tanto, se trata de un problema real que, probablemente, se está subestimando, ya que las muestras para gasometría se procesan sin centrifugar, lo que hace que resulte imposible identificar visualmente la presencia de concentraciones elevadas de sustancias interferentes, como la hemoglobina libre de células, la bilirrubina y los lípidos, lo que puede generar un sesgo sustancial en muchos de los parámetros que se miden con los analizadores modernos de gases en sangre. En este contexto, no se pueden utilizar los procedimientos habitualmente empleados en los laboratorios clínicos para corregir o mitigar la interferencia causada por la lipemia (p.ej. precipitación con soluciones de limpieza, disolución y ultracentrifugación), ya que la mayoría de las gasometrías se realizan fuera del laboratorio y los analistas desconocen que, cuando las muestras se procesan con sangre total, pueden presentar una concentración de lípidos elevada. Además, en caso de contaminación por fluidos, dichos procedimientos podrían no resultar eficaces a la hora de resolver el efecto dilucional, por lo que sería más apropiado indicar que se rechacen las muestras, ante cualquier sospecha de contaminación por la administración de fluidos intravenosos, tanto en el laboratorio central como en las pruebas a pie de cama (POCT).

En un estudio previo, Jara-Aguirre y col [9] reunieron muestras de gasometría arterial con heparina de litio residual a los 60 minutos de la extracción con emulsión lipídica al 20 % de nutrición parenteral total (contaminación de la sangre de entre el 0 y el 15 %) y, a continuación, midieron una serie de parámetros en un analizador de gasometría arterial Radiometer ABL90 FLEX (Radiometer Medical ApS, Dinamarca). Se comprobó que se superaban los criterios de error aceptables cuando la contaminación de la emulsión lipídica era ≥1 % para pH (reducción), pO_2_ (aumento) y pCO_2_, (reducción), cuando la contaminación era ≥5 % para sodio (disminución) y potasio (aumento), y ≥10 % para iCa^2+^ (aumento) y tHb (disminución), respectivamente. A diferencia de este estudio previo, en el que se reunieron y procesaron las muestras más de una hora después de la extracción, en nuestro estudio, empleamos muestras individuales y realizamos todas las gasometrías en los 15 minutos posteriores a la extracción. Esto nos permitió adherirnos a las guías clínicas actuales de la CLSI [8], en las que se establece que las gasometrías deben realizarse en los 30 minutos posteriores a la extracción de la sangre. Por otro lado, empleamos una nueva emulsión de lípidos, diferente a la utilizada por Jara-Aguirre y col (Intralipid, Fresenius Kabi, Uppsala, más antigua, distribuida por Baxter Healthcare Corporation, Deerfield, EE.UU) [9], que ni siquiera está ya disponible en algunos países. Estas importantes diferencias en los diseños de los dos estudios podrían explicar algunas de las discrepancias observadas [9]. De hecho, aunque también observamos una reducción de pCO_2_, sodio y tHb, junto con un aumento de pO_2_, en nuestro estudio, las concentraciones de iCa^2+^ y potasio fueron disminuyendo gradualmente, a medida que aumentaba la contaminación lipídica. Independientemente de estas diferencias, que se podrían atribuir a la composición de la solución lipídica, la evidencia más relevante aportada por estos dos estudios es que se pueden producir desviaciones significativas en las concentraciones de gases en sangre en la jeringa cuando la sangre está contaminada con una solución lipídica, lo que puede derivar en un error diagnóstico y un tratamiento inapropiado.

En lo que respecta a los resultados obtenidos en nuestro estudio, la variación de algunos analitos fue predecible, ya que las desviaciones observadas se debieron fundamentalmente al efecto dilucional de la solución lipídica. Esto es especialmente aplicable a las concentraciones reducidas de sodio, cloruro, iCa^2+^, tHb, glucosa, Hct y tHb. Por otro lado, las variaciones en potasio merecen especial atención. Cabe señalar que, aunque se produjo una reducción de su concentración en las dos jeringas contaminadas (−5,2 % y −5,3 %, respectivamente), la reducción que se observó en la jeringa con lípidos al 10 % fue inferior a la esperada, debido únicamente al efecto dilucional. De este modo, otros factores podrían haber contribuido a la desviación observada en la concentración medida de dicho analito, siendo la hemólisis *in vitro* la explicación más probable. Esto concuerda con el hecho de que observamos un aumento dependiente de lípidos en el índice H en plasma centrifugado, lo que refleja la presencia de daño eritrocitario (osmótico) y descomposición, probablemente debido a la concentración extremadamente elevada de lípidos en las muestras [15]. Este hallazgo no debe sorprender, ya que Dimeski y col demostraron hace casi 20 años que la hiperlipidemia (especialmente la hipertrigliceridemia) puede inducir hemólisis *in vitro* [16]. En cuanto a las variaciones en los gases en sangre, podemos descartar la posible presencia de artefactos provocados por la manipulación de las muestras ya que, a diferencia del estudio de Jara-Aguirre y col. [9], no añadimos lípidos a las muestras residuales de gases en sangre, sino que la jeringa de gases sanguíneos se llenó previamente con la solución lipídica, antes de extraer la sangre, lo que minimizó el riesgo de introducir burbujas de aire. De este modo, la explicación más plausible es que la hiperosmolaridad sostenida provocada por concentraciones muy elevadas de triglicéridos en las muestras podrían haber provocado hipoxia eritrocitaria y un cambio hacia el metabolismo anaeróbico, lo que se asocia a un menor consumo de oxígeno y una mayor generación de lactato [17], tal como se observó en nuestro estudio.

Una posible limitación de este estudio podría ser que empleamos sangre venosa para evitar las molestias de una punción arterial en nuestra cohorte de voluntarios aparentemente sanos. Otra limitación es el hecho de que la solución lipídica *in vitro* no reproduce fielmente la hiperlipidemia *in vivo*. Sin perjuicio de lo anterior, nuestros hallazgos indican que la hiperlipidemia artefactual (principalmente la hipertrigliceridemia) causada por la contaminación de lípidos exógenos puede tener un impacto clínicamente significativo en el análisis de gases en sangre. Dado que identificar la lipemia dentro de una jeringa de gasometría resulta difícil, animamos encarecidamente a los fabricantes de analizadores de gasometría a que se comprometan a desarrollar nuevos instrumentos equipados con los índices séricos. Aunque la estrecha relación entre la dilución y las variaciones en la mayoría de los analitos observada en nuestro estudio debería descartar la posibilidad de interferencias analíticas, no pudimos excluir que la elevación del índice hemolítico, el lactato sanguíneo o la presión parcial de los gases se pueden haber producido a causa de posibles interferencias.
